# The Usher gene cadherin 23 is expressed in the zebrafish brain and a subset of retinal amacrine cells

**Published:** 2012-09-05

**Authors:** Greta Glover, Kaspar P. Mueller, Christian Söllner, Stephan C.F. Neuhauss, Teresa Nicolson

**Affiliations:** 1 Oregon Hearing Research Center and Vollum Institute, HHMI/Oregon Health & Science University, Portland, OR; 2 Institute of Molecular Life Sciences, University of Zurich, Zurich, Switzerland; 3 Max Planck Institute for Developmental Biology, Tübingen, Germany

## Abstract

**Purpose:**

To characterize the expression pattern of cadherin 23 (*cdh23*) in the zebrafish visual system, and to determine whether zebrafish *cdh23* mutants have retinal defects similar to those present in the human disease Usher syndrome 1D.

**Methods:**

In situ hybridization and immunohistochemistry were used to characterize *cdh23* expression in the zebrafish, and to evaluate *cdh23* mutants for retinal degeneration. Visual function was assessed by measurement of the optokinetic response in *cdh23* siblings and mutants.

**Results:**

We detected *cdh23* mRNA expression in multiple nuclei of both the developing and adult central nervous system. In the retina, *cdh23* mRNA was expressed in a small subset of amacrine cells, beginning at 70 h postfertilization and continuing through adulthood. No expression was detected in photoreceptors. The *cdh23*-positive population of amacrine cells was GABAergic. Examination of homozygous larvae expressing two different mutant alleles of *cdh23—cdh23^tc317e^* or *cdh23^tj264a^—*revealed no detectable morphological retinal defects or degeneration. In addition, the optokinetic response to moving gratings of varied contrast or spatial frequency was normal in both mutants.

**Conclusions:**

Unlike in other vertebrates, *cdh23* is not detectable in zebrafish photoreceptors. Instead, *cdh23* is expressed by a small subset of GABAergic amacrine cells. Moreover, larvae with mutations in *cdh23* do not exhibit any signs of gross retinal degeneration or dysfunction. The role played by *cdh23* in human retinal function is likely performed by either a different gene or an unidentified *cdh23* splice variant in the retina that is not affected by the above mutations.

## Introduction

Usher syndrome (USH) is an autosomal recessive human disease characterized by varying degrees of hearing loss, vestibular dysfunction, and progressive loss of vision (retinitis pigmentosa). The most severe form, Usher syndrome type 1 (USH1), is caused by a mutation in one of seven genes. USH1 patients are profoundly deaf from birth, and in general have an earlier onset of visual symptoms compared to the less severe clinical subtypes, USH2 and USH3 [1,2]. Of the seven genes known to cause USH1, five have been identified: myosin 7a (USH1B), harmonin (USH1C), cadherin 23 (USH1D), protocadherin 15 (USH1F), and SANS (USH1G) [3]. The characterization of USH genes has led to the proposal that many of the proteins they encode interact within the specialized ciliary structures of the hair cell and the photoreceptor, the respective sensory receptor cells of the auditory/vestibular and visual systems [3–6].

Mouse models of USH have been especially useful in revealing the function of many of the USH genes in the auditory/vestibular system. Mice with mutations in the five USH1 genes all have severe defects in hearing and balance, accurately recapitulating the human disease symptoms [7]. Surprisingly, the visual component of USH1 is not recapitulated in the mouse. While protein expression in the photoreceptor or adjacent pigment epithelium has been demonstrated for the majority of USH1 proteins, evidence of blindness and retinal degeneration has not been found in mutant mice [6]. Perhaps the best-characterized example of this failure to phenocopy the human disease is the mouse model for USH1D, with mutations in cadherin 23 (*CDH23*). Like the other USH mouse models, *Cdh23* mutants have been successfully used to understand the deafness component of this disease, showing that CDH23 makes up part of the tip link in sensory hair cells, a structure that is crucial for the proper mechanotransduction of sound waves [8,9]. In the retina, *Cdh23* is expressed in mouse photoreceptors [10–12]. Some slight abnormalities in the electroretinograms (ERGs) of mutants have been found in select alleles [13]; however, no retinal degeneration has been observed.

Several explanations for the comparatively mild visual phenotype in mice versus humans have been proposed. First, another protein may substitute for CDH23 in mouse, but not human, photoreceptors. The most likely candidate is protocadherin 15 (PCDH15), but double homozygous *Pcdh15/Cdh23* mouse mutants do not show retinal defects [14]. Second, species differences in lifespan or intensity of light exposure could prevent detection of disease symptoms. Third, species differences in alternative splicing could underlie retinal-specific insensitivity to all the known mouse *waltzer* mutations [10,14].

Like the mouse USH1 models, several zebrafish lines with mutations in USH1 genes also exhibit profound deafness and balance defects, including myosin 7a (*myo7a)*, *pcdh15a*, and *cdh23* [15–17]. Transcripts for *myo7a* have been found in hair cells, but there are no published reports of expression in the retina. The *pcdh15a* gene is not expressed in retina except at very early stages, although its paralog, *pcdh15b*, is expressed in photoreceptors during larval retinal development [16]. Additionally, knockdown of *pcdh15b* causes both photoreceptor degeneration and a significant reduction in ERG amplitude [17]. Thus, visual defects are possible in zebrafish mutants, although in this particular case, the *pcdh15* gene was duplicated during evolution. In the case of *cdh23*, only one copy is present in the zebrafish genome. We sought to explore the expression and function of *cdh23* in the zebrafish visual system, and to determine the suitability of zebrafish *cdh23* mutants as a model for the retinal component of USH1D.

In this study, we describe a unique retinal expression pattern of *cdh23* in a very small subset of amacrine cells rather than photoreceptors. In addition, we characterize the expression pattern in the brain, where *cdh23* is expressed in multiple regions beginning very early in brain development. We found that larvae homozygous for either of two different *cdh23* mutations have morphologically normal retinas and normal visual function (assessed by measuring the optokinetic response, OKR), despite both having severe deafness and balance defects. The absence of *cdh23* expression in zebrafish photoreceptors indicates distinct functions of CDH23 in the human and zebrafish retinas. The timing and expression pattern of *cdh23* in the zebrafish brain and retina predicts a role in circuit formation during neural development, as well as in maintenance of connections in the mature nervous system.

## Methods

### In situ hybridization

Wholemount in situ hybridization was performed essentially as described in The Zebrafish Book, using a digoxigenin-labeled probe generated using a digoxigenin labeling kit (Roche, Penzberg, Germany). Probes were synthesized from *cdh23* fragments cloned into pcR2.1 TOPO (Invitrogen, Carlsbad, CA). The 5′ antisense probe consisted of nucleotides 448–1,566 from *cdh23* cDNA, while the 3′ antisense probe contained nucleotides 9730–10,515. For in situ hybridization followed by immunostaining on cryosections, the temperature of incubation was reduced from 63 °C to 56 °C. Images of in situ hybridization were taken on a Zeiss AxioImager.M1 wide-field microscope using a 10× or 20× dry objective. Images were acquired with an AxioCam MRc5 color digital camera using Axiovision software, and subsequently adjusted for contrast in Photoshop CS2 (Adobe, San Jose, CA) using the levels, curves or brightness/contrast adjustment functions.

### Immunohistochemistry

Zebrafish were fixed in 4% paraformaldehyde in PBS overnight, cryoprotected in increasing concentrations of fish skin gelatin/sucrose in PBS, ending in 25% gelatin, 15% sucrose, and then frozen in blocks and cut into 14 µm sections on a Leica CM180 cryostat. Immunostaining was performed using standard on-section immunohistochemistry protocols. Permeabilization of sections was achieved by including 0.02% saponin (S7900; Sigma, St. Louis, MO) in all block, wash, and antibody incubation solutions. Antibodies and dilutions were as follows: Ab5E11 (mouse, 1:500, gift from J. Fadool, Florida State University, Tallahassee, FL), calretinin (rabbit; 1:5,000; Swant [Swiss Antibodies], Marly, Switzerland), FMRF (rabbit, 1:100; Phoenix Pharmaceuticals, Burlingame, CA), glutamate decarboxylase (GAD65/67) rabbit; 1:500; Abcam, Cambridge, MA), parvalbumin (mouse; 1:500; Millipore, Billerica, MA), serotonin (rabbit; 1:250; Sigma), acetylated tubulin (mouse; 1:2000; Sigma), tyrosine hydroxylase (TH; mouse; 1:500; Immunostar, Hudson, WI), Cy3 antimouse and Cy3 antirabbit (1:1,000; Jackson Immunoresearch, West Grove, PA). Images of immunostained cryosections were acquired on either an Olympus Fluoview (Olympus America Inc., Center Valley, PA) or Zeiss LSM710 (Carl Zeiss AG, Oberkochen, Germany) confocal microscope, and processed for display using ImageJ [18] and Adobe Photoshop. ImageJ version 1.43u was used to convert files to tif format and to produce color overlays. Photoshop CS2 (Adobe) was used to adjust contrast using curves, levels and/or brightness/contrast image adjustment functions.

### Eye injections

Serotonin (H7752; 100 mM; Sigma) in embryo medium was injected intraorbitally using a protocol modified from [19,20]. Briefly, glass microinjection pipettes were pulled on a Nashirige PC-10 (Nashirige International USA, Long island, NY) and filled with 100 mM serotonin (5-hydroxytryptamine, 5-HT) and 100 mM ascorbic acid in embryo medium. The tip of the glass micropipette was broken with forceps to a diameter sized to slip between the edge of the lens and retina without causing excessive damage. Approximately 50 nl was injected into the left eye of 4 days postfertilization (dpf) larvae anesthetized in Tricaine and mounted in 2% low-melt agarose; the right eye was left uninjected to control for nonspecific immunostaining. Following injection, larvae were transferred to fresh embryo medium and allowed to recover at 28 °C for 3 h, and were then fixed in 4% paraformaldehyde overnight at 4 °C. Following this, they were processed for in situ hybridization and subsequent antiserotonin immunohistochemistry.

### Detection of splice variants

Total RNA was isolated from wild-type zebrafish eyes or enucleated larvae (remaining tissue following the removal of eyes) by homogenization of the larvae in TRIZOL reagent (Invitrogen, Life Technologies, Grand Island, NY) using a 25 gauge needle, followed by RNeasy purification (Qiagen, Valencia, CA). Reverse transcription was performed with the Superscript III First Strand cDNA synthesis kit (Invitrogen) using oligo(dT) primers. PCR products were subcloned into the pCRII-TOPO vector (Invitrogen) and sequenced. The splice variants were confirmed at least once with another independent PCR. Primer sets were as follows (written 5′ to 3′). Nested primers to amplify the region from extracellular cadherin repeat, or ectodomain 14 (EC14) to the 3′ UTR were as follows. External primers: 5′-TGT GGA CCG CTA CCT GCT TAA AGT-3′ 5′-TGA GGC ATT CAG AGT CCA CAC ACA-3′, internal primers: 5′-TCA GTG AGA ATG TGG GTG GTG GAA-3′ 5′-GTG TCG CTG CCT TTG TTT CTG TGT-3′. Primers to amplify the cytoplasmic domain: 5′-CTG AAG GCA GTA GTT GCA GGC T-3′ 5′-TCA TAA CTC TGT GAT CTC TAA CGG AC-3).

### Optokinetic response

As a test for visual function, the OKR of 5 dpf homozygous larvae of both alleles, as well as their wild-type siblings, was measured as previously described [21]. In brief, larvae were embedded in 3% prewarmed methylcellulose, aligned to lay dorsal side up and placed in the center of a white paper drum (d=9 cm). Vertical black-and-white sine-wave gratings of varying spatial frequency and contrast were projected onto the inside of the drum [22]. The grating pattern was rotated around the larva with an angular velocity of 7.5 °/s. To minimize the frequency of saccades, the direction of motion was altered with a frequency of 1/3 Hz.

Eye movements were recorded at 5 frames per second by a CCD camera attached to a dissecting microscope. Custom-made software based on NI LabView 7.1 and NI-IMAQ 3.7 (National Instruments, Austin, TX) was used to extract the orientation of each eye in real time. This software is now licensed to TSE (Homburg, Germany), under the name VisioTracker. Eye velocity was calculated as the first derivative of the orientation with respect to time. Saccades were filtered out from raw measurements of eye velocities by applying a fixed threshold of 20 °/s. The resulting curves of slow-phase velocities were smoothed by a running average with a sliding window of seven frames [23]. Finally, eye velocities were averaged over frames with identical stimulus properties, as well as for the two eyes of each larva. Graphs were generated using R 2.9.2.

Zebrafish were cared for according to the guidelines set forth by the Institute for Laboratory Animal Research.

## Results

**Figure 1:**
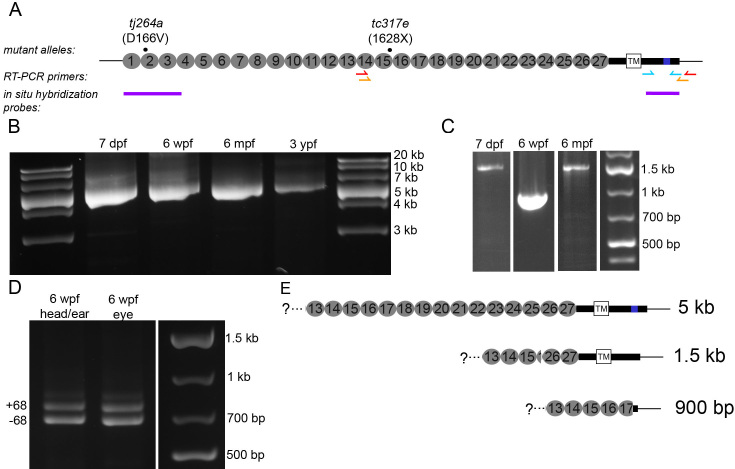
Presence of full-length and splice variants of *cdh23* mRNA in zebrafish larval and adult retina. **A**: Schematic illustrating the locations of the mutations in the two *cdh23* mutant fish lines, reverse transcriptase (RT)–PCR primers, and the 5′ and 3′ in situ hybridization probes used in this study. For the RT–PCR primers, the red and orange arrows indicate the location of the nested primer set used to amplify *cdh23* in panels **B** and **C**, while the cyan arrows indicate the primer set used in **D**. **B**: Developmental time-course showing the full-length *cdh23* RT–PCR product isolated from eyes of various ages (n=3). **C**: Smaller RT–PCR products were also present. A shorter variant containing the transmembrane (TM) segment was amplified from 7 days postfertilization (dpf) and 6 months postfertilization (mpf) retinal transcripts (n=2). A short, soluble variant was amplified from 6 weeks postfertilization (wpf) eyes (n=2). Neither shorter variant was isolated from 3 years postfertilization (ypf) eyes (n=2). **D**: Using primers directed against sequence encoding the entire C-terminus, both the full-length version (containing exon 68) and the version present in the shorter variant containing the TM segment were isolated from eye or enucleated head (n=2). **E**: Schematics depicting the sequence and expected size of the full-length and two short forms of *cdh23* isolated in (**B**) and (**C**).

Previous studies have shown a critical function of *cdh23* in zebrafish hair cells by characterizing *cdh23* mRNA (mRNA) expression and function in wild-type and *cdh23* mutant fish [16,24,25]. In the present study, we focused on characterizing *cdh23* alternative splicing, expression in the brain and retina, and the effect of *cdh23* mutations on the visual system. Figure 1A summarizes the tools used in this study. The positions of the mutations in the two *cdh23* alleles are shown. The *cdh23^tc317e^* allele contains a t>g point mutation three nucleotides before the splice acceptor site at the exon 38 junction. This creates a novel splice acceptor site, resulting in an ag insertion between exon 37 and 38, leading to a frameshift resulting in a stop codon at amino acid 1628, within the 15th extracellular repeat or ectodomain (EC15). This site is present in all three splice forms. The *cdh23^tj264a^* allele is a point mutation resulting in a single amino acid substitution, D166V, within EC2. Both mutations exhibit severe defects in hearing and balance; homozygous mutant larvae show the complete absence of a startle response, as well as strong circling behavior, which is typical of mutants with vestibular defects. The length and position of the two in situ hybridization probes, and the positions of the primers used to amplify *cdh23* and its splice variants in the eye, are also indicated in Figure 1A.

### Expression of *cdh23* transcripts in zebrafish eye

To initiate our study of the role of *cdh23* in zebrafish visual function, we characterized its expression in the developing zebrafish eye by reverse transcriptase (RT)–PCR (Figure 1). To describe the splice isoforms present specifically in zebrafish eyes, we performed RT–PCR on dissected eyes isolated from zebrafish at various stages of development. Using nested primers directed against EC14 and the 3′UTR (Figure 1A), we found that, in contrast to the case in mice, zebrafish eyes express the full-length isoform at all developmental stages examined: larval (7 dpf), juvenile (6 weeks postfertilization [wpf]), adult (6 months postfertilization [mpf]), and end-of-life (3 years postfertilization [ypf]; Figure 1B). Alternative splicing of *cdh23* has been studied extensively in the mouse, and the presence of shorter *cdh23* splice variants has been implicated as one explanation for the lack of visual phenotype in mouse *cdh23* mutants [10]. Using primers similar to those used in our study, Söllner et al. [16] amplified two additional splice variants from whole larvae: a short transmembrane (TM) isoform that is missing EC15–25 (and is also missing exon 68), and a soluble isoform that skips from EC17 to a short sequence in exon 69, the final exon. We therefore investigated the possibility that eye-specific variants are present in the zebrafish. We were able to amplify the short TM isoform from 7 dpf and 6 mpf eyes, and the soluble isoform from 6 wpf eyes (Figure 1C). We were unable to amplify either shorter variant from 3 ypf eyes. Using primers specific to the region encoding the cytoplasmic domain (Figure 1A), both +exon68 and -exon68 variants of *cdh23* were amplified from zebrafish eyes and enucleated larvae at 7 dpf (not shown) and 6 wpf (Figure 1D). Only the +exon68 form was amplified with the EC14–3′ UTR primer pairs described above. We do not know the biologic relevance of these two shorter variants, although both would be affected by the *cdh23^tc317e^* mutation. However, it is possible that there are additional splice variants present that we did not detect.

### Expression pattern of *cdh23* in the developing zebrafish brain and retina

**Figure 2:**
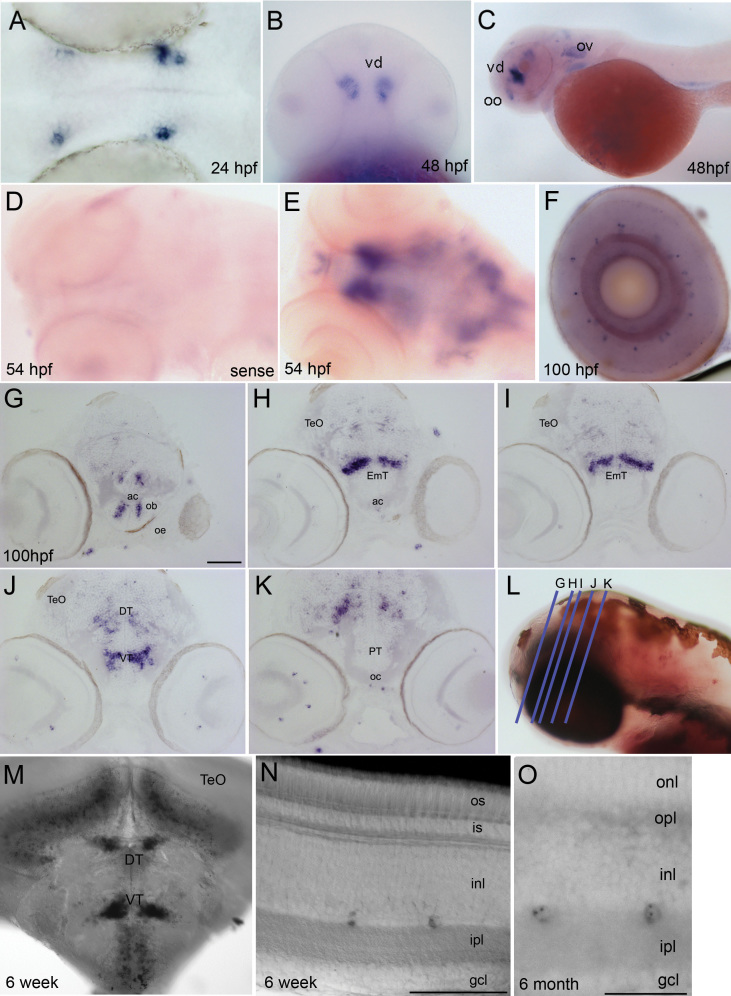
*Cdh23* transcripts are expressed in the zebrafish brain and retina. In situ hybridization showing *cadherin 23* mRNA (mRNA) expression in the developing and mature zebrafish nervous system. **A**: Dorsal view of a zebrafish brain at 24 h postfertilization (hpf); the blue/purple signal shows the first detectable expression of *cdh23* mRNA in two paired nuclei in the diencephalon, adjacent to the eye. **B**: Ventral view of intensely stained deep nuclei in the ventral diencephalon (vd) at 48 hpf. **C**: Lateral view of 48 hpf larva. The two most prominent nuclei labeled are located in the olfactory organ (oo) and the ventral diencephalon. Additional brain labeling is also observed, as is labeling of hair cells in the otic vesicle (ov). **D**: Sense control shows absence of nonspecific labeling. **E**: Dorsal view of the brain at 54 hpf, showing telencephalic and ventral diencephalic paired nuclei from a different angle, as well as additional dispersed cells in the mesencephalon and rhombencephalon. **F**: In the eye, a very small subset of inner layer cells is labeled. **G**-**K**: Post–in situ hybridization cryosectioning allowed the more accurate identification of labeled brain nuclei. Paired telencephalic nuclei were located just medial to the olfactory bulb (ob), in the subpallium (**G**). Caudal to the anterior commissure (ac), very intense labeling was found in bilateral bar-shaped structures in the eminentia thalami (EmT). **H**, **I**: Continuing caudally (**J**), bar-shaped nuclei became more globular in the ventral thalamus. Scattered labeling was also observed in the dorsal thalamus and points caudal in **J**-**K**. **L**: Blue lines represent planes of cryosections made in **G**-**K**. **M**: *Cdh23* in situ hybridization in 6 weeks postfertilization (wpf) zebrafish shows ventral and dorsal thalamic labeling similar to that seen in larval stages, but is expanded to even more ventral areas, and to cells lying just ventral to the optic tectum (TeO). **N**, **O**: At 6 wpf and 6 months postfertilization (mpf), retinal labeling is still restricted to a very small subpopulation of amacrine cells. gcl, ganglion cell layer; inl, inner nuclear layer; ipl, inner plexiform layer; is, inner segment; onl, outer nuclear layer; opl, outer plexiform layer; os, outer segment. Scale bars=100 µm (**G**-**K**, **M**), 50 µm (**N**), 20 µm (**O**).

We next characterized the expression pattern of *cdh23* in developing larvae by in situ hybridization (Figure 2). The first detectable staining was observed approximately 18–24 h postfertilization (hpf) in two paired nuclei deep in the diencephalon (Figure 2A,B), and concurrently in the first developing inner ear hair cells (data not shown). By 48–54 hpf, additional nuclei appeared near the olfactory bulb and in the hindbrain (Figure 2C,E). In contrast to the expression pattern observed for *pcdh15b* [17], we did not detect the *cdh23* transcript in retinal photoreceptors. Instead, a very small subset of amacrine cells was positive for *cdh23* mRNA starting at 70 hpf (Figure 2F). Approximately 30 amacrine cells/retina were labeled, as counted in transverse 14 µm serial cryosections through the entire retina (average of six retinas, data not shown). Labeling with either 5′ or 3′ antisense probes produced identical results; staining patterns were indistinguishable in *cdh23^tc317e^* siblings (mixed wild-type and heterozygous genotypes) and homozygous mutants (shown for the retina in Appendix 1). The images in all other figures show results using the 5′ antisense probe, which exhibited more robust labeling. No staining was observed using a sense control probe (Figure 2D).

We generated serial cryosections following in situ hybridization of whole larvae to examine the brain structures expressing *cdh23* in more detail (Figure 2G-L). Expression of *cdh23* in the brain was localized to the subpallium, eminentia thalami, ventral thalamus, sparse cells in dorsal thalamus, and scattered cells throughout hindbrain. Of the brain nuclei labeled, none were obvious visual centers, although the subpallium and thalamic nuclei have multiple inputs and outputs, and could therefore participate in visual signal processing [26,27]. The *cdh23*-labeled hindbrain neurons were not concentrated in a single nucleus, but instead were scattered throughout the hindbrain. Specific brain nuclei were identified by consulting a zebrafish brain atlas [28] and by performing on-section immunocytochemistry following in situ hybridization for the following antigens, whose localization has previously been described: acetylated tubulin, TH, FMRF, and calretinin (data not shown). To determine whether or not this expression pattern was maintained in older fish, we performed in situ hybridization on transverse vibratome sections from 6 wpf brains and retinas, as well as 6 mpf retinas. In the 6 wpf brain, the *cdh23*-positive nuclei evident in the larval stages were still labeled (Figure 2M and data not shown); additional cell populations were also labeled in the optic tectum and subthalamic area. As in the larval retina, a sparse subpopulation of amacrine cells, but not photoreceptors, were *cdh23* positive in both 6 wpf and 6 mpf retinas (Figure 2N,O).

### Identification of the subtype of *cdh23*-expressing amacrine cells

**Figure 3:**
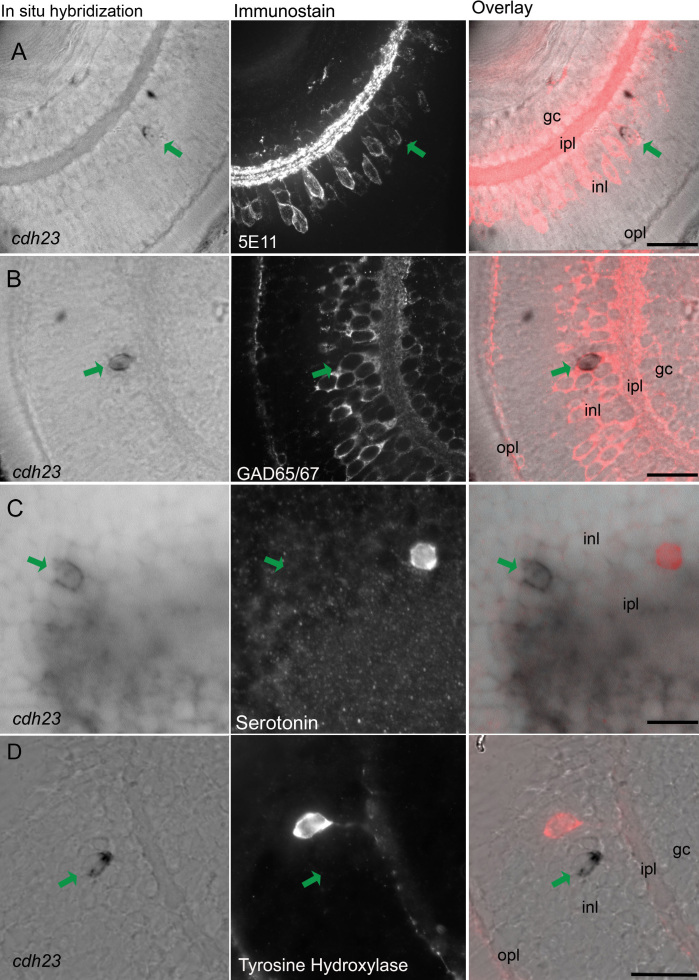
*Cdh23* is expressed in GABAergic amacrine cells. In situ hybridization of *cdh23* was combined with immunocytochemistry for various amacrine cell markers. In each row of images, the left panel shows retinal slices labeled with the *cdh23* mRNA (mRNA) probe, the center panel shows fluorescent immunostaining, and the right panel shows an overlay of the in situ hybridization and the immunofluorescence images. **A**: *cdh23*-positive retinal cells colabel with the pan-amacrine cell antibody 5E11. **B**: *cdh23*-positive amacrine cells also colocalize with an antibody to the GABAergic cell marker glutamate decarboxylase 65/67 (GAD65/67). **C**, **D**: Neither serotonin-positive nor tyrosine hydroxylase (TH)-positive amacrine cells colabel with *cdh23* mRNA. Scale bar, 20 µm.

To further explore the identity of *cdh23*-positive amacrine cells, we combined in situ hybridization for *cdh23* mRNA with immunohistochemistry for various amacrine cell markers. When determining colocalization of an amacrine cell marker with *cdh23* mRNA labeling, this technique gave better single-cell resolution compared to two-color in situ hybridization. Attempts to develop a reliable antibody to zebrafish Cdh23 were unsuccessful; therefore, a simple double immunostain was not feasible. By reducing the stringency of our whole mount in situ hybridization and washing conditions, we were able to preserve immunogenicity of several antigens. Using this approach, we determined that the *cdh23*-positive cells were indeed amacrine cells as shown not only by morphology and position, but also by colocalization with the amacrine cell–specific antigen 5E11 [29] (Figure 3A). Most amacrine cells are either GABAergic or glycinergic [30]. Immunostaining for the GABAergic cell marker glutamate decarboxylase 65/67 (GAD65/67) showed that *cdh23*-positive amacrine cells were invariably GABAergic (Figure 3B). Many different GABAergic amacrine cell subclasses have been further defined based on the presence of a second molecular marker. We next examined colocalization of *cdh23* with antigens known to define subtypes of GABAergic amacrine cells.

Two GABAergic amacrine populations in zebrafish are known to be present at a density very similar to that which we observed for *cdh23*, and therefore warranted investigation. First, a serotonin transporter (SERT)–positive amacrine population has been described in zebrafish [31]. While it has not been extensively characterized in zebrafish, this cell may be similar to the A17 amacrine described in other organisms [32–35]. The A17, or indolamine-accumulating amacrine cells, express a SERT, though these cells do not accumulate serotonin (5-HT) under normal conditions [36]. By exploiting the presence of this SERT, the cell type can be labeled by allowing the uptake of exogenously applied 5-HT, followed by immunostaining with an anti-5-HT antibody [37]. Using a protocol modified for zebrafish larvae, we performed intraocular injection of 5-HT in live larvae, then processed the larvae for in situ hybridization and anti-5-HT immunostaining. None of the *cdh23*-positive cells colabeled with anti-5-HT (Figure 3C). A second sparse GABAergic amacrine population described in zebrafish is the dopaminergic interplexiform cell [38–40]. As was the case for the 5-HT-positive population, *cdh23* mRNA did not localize to cells labeled by an antibody to TH, a marker for dopaminergic amacrine and interplexiform cells. We also compared *cdh23* in situ labeling to a *pax6* promoter-driven GFP transgenic line [41]. The cells labeled by GFP in this line are of at least two types: the parvalbumin-positive type and the ChAT-positive type. No colocalization of GFP and *cdh23* in situ label was observed (Appendix 2). In addition, *cdh23*-positive amacrine cells did not colabel with antibodies to endogenous calretinin (Appendix 2) or parvalbumin (data not shown). We were therefore unable to positively identify a secondary marker for the GABAergic, *cdh23*-positive amacrine cell population.

While these amacrine cells were of great interest considering the role of *cdh23* in vision, we were also struck by the intense labeling in the deep diencephalic paired nuclei. Robust labeling was detectable both very early in development, as well as in adult brain. Overall, these cells comprised the most intensely labeled brain region labeled by *cdh23* RNA probes. Double immunofluorescence/in situ hybridization experiments showed that a subset of these diencephalic, *cdh23*-positive cells were also positive for TH (Appendix 2). Of the three clusters of dopaminergic diencephalic neurons present at this age [42], it was those of the ventral thalamus (ventral tegmentum) that partially colocalized with neurons in the more caudal regions of the *cdh23*-positive deep diencephalic nuclei. The fibers of these ventral tegmental dopaminergic neurons can be followed ventrocaudally, where they join a dense network of TH-positive fibers that project in four different directions [42]. The more rostral *cdh23*-positive cells in this region, those clustered in a striped pattern, were situated just caudal and dorsal to the anterior commissure, but ventral to the calretinin-positive pretectal cluster [43], and were not TH-positive (data not shown).

To identify the projection pattern of *cdh23*-positive cells in the retina and brain, we tested multiple Cdh23 antisera, including several new antibodies that we generated against the zebrafish Cdh23 sequence. Unfortunately, each Cdh23 antibody produced nonspecific staining using multiple staining protocols in whole mount and cryosectioned brain and retinal tissue. To find an alternative means by which to identify the projection pattern of *cdh23*-expressing cells, we generated reporter constructs containing *cdh23* upstream regulatory elements driving GFP. We found that a 6 kb fragment (encompassing the entire 5′ UTR and some surrounding intronic sequence) drove GFP expression in hair cells of the ear and neuromasts (data not shown). By adding the intronic sequence (1 kb, 3 kb or 5 kb) directly upstream of the start codon to the 6 kb 5′ UTR construct, expression in the eye and brain was also observed. The GFP expression pattern, however, was the same for all three constructs: Labeling in the brain was too low to visualize neurites, and in the retina, mainly glycinergic rather than GABAergic amacrine cells expressed GFP (data not shown). Further efforts will be necessary to characterize the *cdh23* promoter in the brain and retina.

### Retinal morphology in *cdh23* siblings and mutants

**Figure 4:**
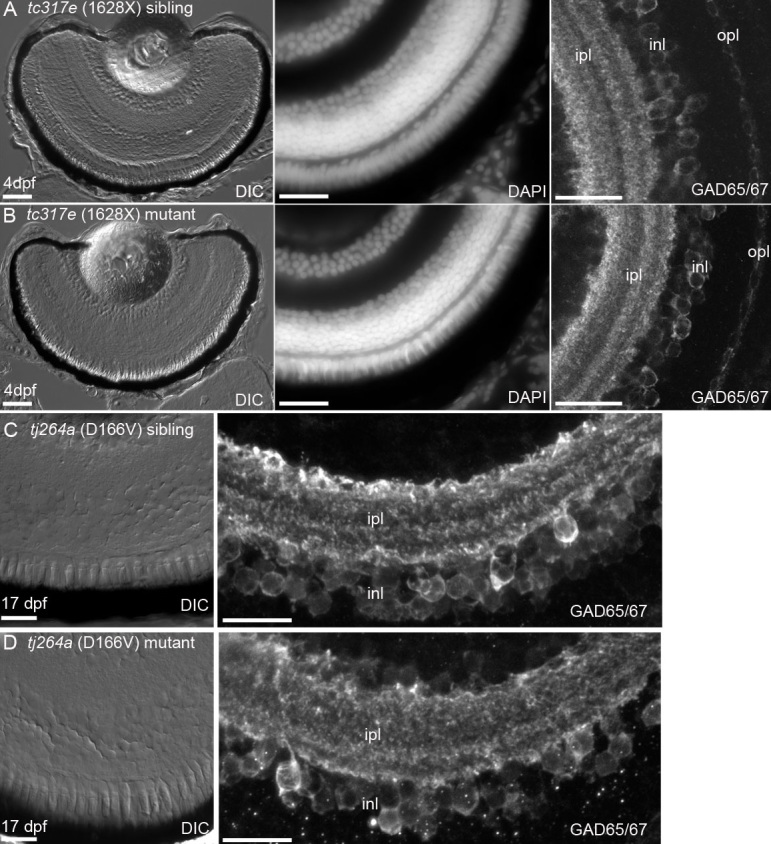
Retinal morphology is normal in c*dh23* mutants. Zebrafish lines carrying one of two different severe *cdh23* mutant alleles, which each cause profound deafness/balance defects, were examined for retinal defects. **A**, **B**: Examination of 4 days postfertilization (dpf) retinal cryosections by differential interference contrast microscopy (left panel), by staining with the nuclear dye DAPI (middle panel), or by examining GABAergic amacrine cell morphology and the inner nuclear layer banding pattern with anti-GAD65/67 (right panel) showed that *cdh23^tc317e^* siblings (**A**) and homozygous mutants (**B**) had morphologically identical retinas. **C**, **D**: Siblings and mutants of the *cdh23^tj264a^* allele were raised to 17 dpf and their retinas were examined as in **A**, **B**. No differences in morphology between siblings and mutants were observed at this later stage of development. Scale bar=50 µm in **A**, left panel (applies to **A** and **B** left panels). Scale bar=20 µm for middle, right panels in **A**, **C** (also applies to image directly below).

Retinal morphology was examined at 4 dpf and 17 dpf for two different alleles of *cdh23* mutants. For homozygous larvae carrying either allele, the ability to feed properly is severely compromised, and mutant larvae typically die by 8–9 dpf. This early death significantly limits the developmental stage at which we are able to examine retinal degeneration. With excessive feeding and care, however, mutant larvae were kept alive until 17 dpf, at which point similar-sized siblings and mutants were fixed and their retinas were examined. Cell density and health, examined by both differential interference contrast microscopy and nuclear labeling with DAPI (Figure 4), were identical in siblings and mutants of both alleles at 4 and 17 dpf (Figure 4A,B shows *cdh23^tc317e^* at 4 dpf, Figure 4C,D shows *cdh23^tj264a^* at 17 dpf). In addition, the layering of GABAergic fibers in the inner plexiform layer was indistinguishable between siblings and mutants (Figure 4, right panels). We predict that the defects caused by these two alleles likely do not contribute to gross retinal degeneration or disorganization, but may instead contribute to subtle defects that would require more careful analysis of the *cdh23*-positive amacrine cell population in isolation.

### OKR is normal in *cdh23* mutants

**Figure 5:**
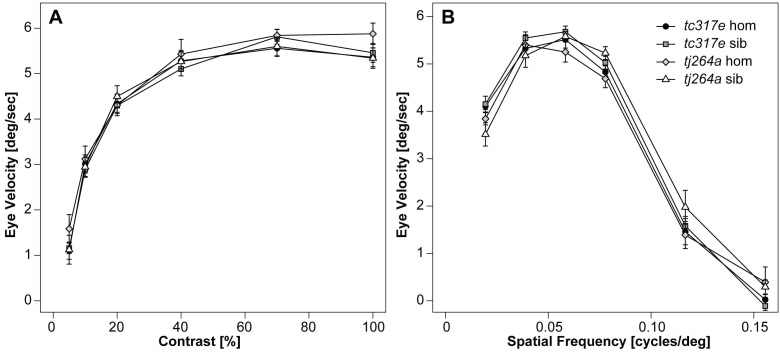
The optokinetic response is normal in both *cdh23^tc317e^* and *cdh23^tj264a^* mutants. Graphs show the averaged slow-phase velocity of both eyes measured under varying contrast (**A**) and spatial frequency (**B**) of the optokinetic stimulation. No significant difference was found between any of the groups (repeated measures ANOVA; contrast: F_3,32_=0.525, p=0.668; spatial frequency: F_3,32_=0.549, p=0.653; n=9 larvae per group).

To investigate whether visual function was affected in *cdh23* mutants, we tested the OKR, varying either the contrast or spatial frequency of the grating or stripes. At 5 dpf, the OKR of homozygous larvae of both alleles was indistinguishable from wild-type responses. We found a reduction neither in contrast sensitivity (Figure 5A) nor in visual acuity (Figure 5B). This result is consistent with an earlier study in which vestibular function was reported to be absent in homozygous *cdh23^tc317e^* (also referred to as *cdh23^1619ag^*) larvae, whereas the OKR occurred under bright light conditions [44]. While we were not able to detect a reduction of the OKR in mutant *cdh23* larvae at this stage of development, testing this particular reflex may not be sufficient to observe functional defects in either the amacrine cells themselves, or upstream in the visual processing centers of the brain.

## Discussion

Mutations in *CDH23* cause a similar phenotype in human, mouse, and zebrafish auditory/vestibular systems. In contrast, the visual defects observed in human USH1D are not recapitulated in the mouse model. In this study, we investigated the role of *cdh23* in the zebrafish visual system, describing both its expression pattern and the visual phenotype of *cdh23* mutants. We observed *cdh23* expression in multiple brain regions and in retinal amacrine cells at both early and late stages of development. Upon examination of retinal tissue and visual behavior of mutant *cdh23* larvae, however, there were no detectable defects in either morphology or visual function, despite the fact that these fish did not respond to acoustic stimuli and had severe balance defects. Overall, zebrafish *cdh23* mutants do not show any of the features of the human retinal phenotype associated with USH1D, and therefore do not provide a good model for the visual component of the human disease. However, characterization of the *cdh23* expression pattern in zebrafish predicts other, as yet undescribed roles for this protein in the retina and brain.

An unexpected result was the absence of *cdh23* expression in the photoreceptors of zebrafish retina, not only because *cdh23* expression has been reported in photoreceptors of both mice and monkeys [10–12,45], but also because the ortholog of a known interaction partner of *cdh23*, *pcdh15b*, is expressed in zebrafish photoreceptors [17]. Recent studies in rodents have established that CDH23 and PCDH15 interact in a heterophilic fashion in the stereocilia of the sensory hair cells in the auditory/vestibular system [8,46]. It has been suggested that this interaction is recapitulated in the photoreceptor layer of the retina, as both CDH23 and PCDH15 have been localized to the connecting cilia [5]. The zebrafish genome contains two copies of *pcdh15*: the first, *pcdh15a*, is important for hearing and balance, while *pcdh15b* is necessary for proper photoreceptor morphology, and hence retinal function [17]. Like *cdh23*, *pcdh15b* is expressed during early zebrafish retinal development, indicating a possible role in cell adhesion as retinal layering is established. Based on the known interaction between CDH23 and PCDH15 in mice, it is perplexing that their expression pattern in the zebrafish retina does not allow for such an interaction. Amacrine cells have been shown to make connections with all types of retinal neurons except for photoreceptors [47,48], making the interaction between Cdh23 and Pcdh15b in the zebrafish retina improbable. It is worth noting that the expression in amacrine cells may not be a peculiarity of the zebrafish retina. In the monkey retina, a CDH23 antibody labels the inner nuclear layer as well as the photoreceptor layer [10]; *CDH23* expression in human and mouse inner retina has not been described.

Our double labeling experiments revealed that *cdh23*-positive amacrine cells express GAD65/67. There are multiple subclasses of GABAergic amacrine cells in the retina, which are defined based on their projection patterns in the inner nuclear layer and/or on their response patterns to visual stimuli. Based on the low number of *cdh23*-positive cells, we predict that this subpopulation will be a wide-field cell, since amacrine cells are thought to evenly tile the retina and therefore process information from the entire visual field [35,49]. There are several wide-field GABAergic amacrine cell subtypes that have been described based on both branching pattern and the presence of a second protein marker. Our data from the combined *cdh23* in situ and amacrine marker immunostains can be used to eliminate several of the known GABAergic wide-field subtypes from consideration for the identity of the amacrine subtype labeled by *cdh23*. First, we can likely eliminate the starburst and the A19 amacrine cells, based on the lack of colocalization between *cdh23* mRNA and calretinin or pax6-GFP ([50] and Webvision). Second, a cell type similar to the A17 indoleamine-accumulating amacrine cell, which is labeled by uptake of serotonin through a serotonin transporter expressed in this cell type, was not positive for *cdh23*. Lastly, TH labels a sparse population of GABAergic wide-field amacrine and interplexiform cells ([38] and Webvision), but TH did not colocalize with *cdh23* mRNA. We were unable to test for colocalization with markers for other GABAergic wide-field cells that contain a second neurotransmitter, like substance P–positive A22 cells, or neuropeptide Y– or cholecystokinin-positive cells (Webvision and [51,52]). Additional amacrine populations have also been described [48]; determining the neurite branching pattern of the *cdh23*-positive amacrine cell will greatly facilitate further characterization of this cell.

The lack of visual defects in *cdh23* mutant larvae could be explained by multiple factors. First, considering that so few retinal cells express *cdh23*, it is likely that the OKR assay is too global a measure of visual function to detect a subtle defect such as might be present in *cdh23* mutant larvae. Once the specific subtype of GABAergic amacrine cells that expresses *cdh23* is determined, it will be possible to design more appropriate behavioral tests to examine the function of this amacrine cell subtype. Similarly, morphological defects present in the retina of fish with a mutation that would affect so few cells would not be easy to detect. There is some evidence that retinal defects originating in amacrine cells can cause detectable morphological abnormalities [53], although these abnormalities were in many cases likely to be caused by defects in the development of a larger population of amacrine cells. Retinitis pigmentosa associated with human USH1D is thought to be caused mainly by defects arising from the photoreceptor cells and the overlying retinal pigment epithelium, and not by amacrine cell degeneration [54]. In some rare cases, however, it may be possible that delayed-onset photoreceptor degeneration could be preceded by disorganization in the inner nuclear layer [55,56].

A second concern regarding the absence of visual defects in zebrafish *cdh23* mutants is the relatively young age at which mutants were examined. Zebrafish mutants with balance defects do not inflate their swim bladders; therefore, prey capture or feeding at the surface of a tank is nearly impossible. Mutant *cdh23* larvae are consequently difficult to raise past the stage when the yolk has been completely reabsorbed; however, with extensive care we were able to increase their life span to about three weeks old. Although zebrafish ERGs and retinal morphology are largely adult-like by 5 dpf, there are aspects of retinal development that occur later, such as acquisition of rod-mediated vision at 15–40 dpf [57]. As it is difficult to raise zebrafish auditory/vestibular mutants past the first week, it is problematic to study late-onset degenerative diseases. In Usher patients, abnormalities in the ERG can be detected as early as 6 months of age, years before the onset of tunnel vision [58]. The ERG of *cdh23* mutants was normal at 7 dpf (data not shown), and the OKR was normal at 5 dpf (Figure 5). Despite the fact that the zebrafish retina has many hallmarks of maturity at these stages, it is possible that examining visual function at later stages would uncover a visual defect.

A third explanation for normal visual function in our zebrafish *cdh23* mutants could be the existence of an unidentified *cdh23* splice variant that may not have been detected by either of our 5′ or 3′ in situ probes. In the mouse, three main forms of *Cdh23* transcript have been identified, encoding two different TM isoforms with either 27 or 7 cadherin repeats, and one short soluble isoform [59]. Additionally, each of these three isoforms has been isolated with two different cytoplasmic domains, either including or excluding exon 68 (the penultimate exon). Evidence for eye-specific splice variants has also been found in the mouse. Lagziel et al. [59] found that the longest *Cdh23* isoform is expressed only in the postnatal mouse inner ear (P0-P10), but not in the retina, indicating that the shorter retinal isoforms would be insensitive to mutations in most of the *waltzer* alleles whose visual function has been studied [13,14]. However, mutations in human *CDH23* that underlie USH1D occur throughout the sequence of the *CDH23* long isoform [60], indicating that human retinal function depends on having a functional full-length *CDH23*. Alternative splicing of *CDH23* in humans has not yet been found [61]. Nevertheless, it could be that an unidentified splice variant is expressed in zebrafish photoreceptors, and by virtue of its alternative splicing pattern, is insensitive to the two mutant alleles used in this study. In general, however, our findings suggest that zebrafish do not make a good model organism for studying the mechanism of retinal degeneration present in human USH1D patients.

While the auditory/vestibular and visual phenotypes define Usher syndrome, there are also multiple reports of mental symptoms associated with the disease [62–64], and brain atrophy has been observed in some Usher patients [65]. It is likely that the expression of *cdh23* that we observe in the zebrafish brain, which is also observed in the mouse [66], is relevant to the human disease as well [67]. Although functional redundancies or brain plasticity seem to eliminate any critical need for CDH23 function in the majority of human USH cases, a subset of patients may exhibit subtle defects in circuit formation or maintenance owing to the loss of CDH23. Further study of CDH23 function in neurons other than sensory hair cells and photoreceptors may reveal additional roles for CDH23 in the nervous system.

## Appendix 1.Expression of *cdh23* mRNA in wild-type and mutant retinas.

To access the data, click or select the words “Appendix 1.” This will initiate the download of a compressed (jpg) archive that contains the file. In situ hybridization using a probe directed against either the 5′- or 3′ region of *cdh23* showed that *cdh23* mRNA (mRNA) is expressed in a subset of amacrine cells of both *cdh23^tc317e^* siblings (left panels) and homozygous mutants (right panels). The 5′ RNA probe used in (**A**, **B**) was complementary to nucleotides 448–1,566 from *cdh23* cDNA. **C**, **D**: The same amacrine cell staining of *cdh23^tc317e^* siblings (**C**) and mutants (**D**) was observed using a 3′ RNA probe complementary to nucleotides 9730–10,515.

## Appendix 2.*Cdh23* mRNA is not expressed in either *Pax6p-GFP*-positive or calretinin-positive amacrine cells.

To access the data, click or select the words “Appendix 2.” This will initiate the download of a compressed (jpg) archive that contains the file. Partial colocalization between tyrosine hydroxylase (TH) and *cdh23* was observed in the ventral tegmentum. **A**: Combined *cdh23* in situ hybridization (left panel) and anti-GFP antibody labeling (middle panel) shows that *cdh23* is not expressed in *pax6p-GFP*-positive amacrine cells (overlay, right panel). **B**: *Cdh23* is also not expressed in calretinin-positive amacrine cells. **C**: In the ventral thalamus, a subset of the bilateral deep diencephalic *cdh23*-positive cells (corresponding to the area shown in Figure 2J) label with anti-TH, which marks the dopaminergic neurons of the ventral tegmentum [42].
